# An ANN-based non-destructive model for age and maturity estimation of the data-deficient Goldblotch Grouper (*Epinephelus costae*): a practical tool for data-poor fisheries in conflict-affected Mediterranean coasts

**DOI:** 10.7717/peerj.21243

**Published:** 2026-05-19

**Authors:** Nader Iskandar Hamwi

**Affiliations:** Department of Animal Production, Faculty of Agricultural Engineering, Latakia University, Latakia, Syria

**Keywords:** *Epinephelus costae*, Artificial Neural Network, Non-destructive aging, Length-at-maturity, Data-poor fisheries, Eastern Mediterranean, Syria

## Abstract

The Goldblotch Grouper (*Epinephelus costae*) is listed as “Data Deficient” by the IUCN, yet it faces intense fishing pressure across the Eastern Mediterranean. In Syria, more than a decade of conflict has dismantled fisheries monitoring infrastructure, leaving managers without essential biological parameters for evidence-based conservation. Traditional methods for age and maturity assessment are destructive, require specialized laboratories, and are infeasible in post-conflict settings. To address this gap, this study presents the first non-destructive artificial neural network (ANN) model to simultaneously estimate age and maturity status using total length as the sole input. The model was trained exclusively on 150 specimens collected from six Syrian coastal sites, Ras al-Bassit to Tartous, during a 12-month period from March 2024 to March 2025. Age was determined by scale reading, and maturity status was assessed by macroscopic examination of gonads after dissection, revealing a contemporary length-at-first-maturity (L_m_ = 30 cm) in Syrian waters. The feedforward multilayer perceptron (MLP) architecture (1–10–2) achieved high predictive accuracy, with a Pearson’s correlation coefficient of *R* = 0.9995 for age estimation (MSE = 0.0091 on the test set), and 100% maturity classification accuracy on the current independent test subset. However, real-world performance may vary in other populations. Our dataset includes individuals up to 74.3 cm and 12 years old, which are substantially larger and older than those reported in recent studies from neighboring regions. This underscores the value of targeted sampling methods, such as speargun, in accessing cryptic, large-bodied spawners. This ANN-based tool provides the first rapid, non-destructive, and field-applicable method for estimating population structure of *E. costae* under data-poor, conflict-affected conditions. By requiring only a length measurement, it offers a potential pathway for real-time decision support in data-poor contexts, supporting science-informed management where traditional monitoring systems have collapsed.

## Introduction

Grouper species (family Epinephelidae) are among the most ecologically and economically valuable fish taxa in the Mediterranean Sea, yet they remain highly vulnerable to overexploitation due to their slow growth, late maturity, site fidelity, and aggregation during spawning (Francour & Pollard, 2018). The Goldblotch Grouper, *Epinephelus costae* (Steindachner, 1878), plays a significant ecological and commercial role in demersal fisheries throughout the Eastern Mediterranean. This species is widely documented in global fish databases such as FishBase (Froese & Pauly, 2025) and taxonomic references including the FAO Species Catalogue (Heemstra & Randall, 1993). Its conservation status is currently assessed as ‘Data Deficient’ (DD) under the IUCN Red List criteria. This status reflects critical gaps in fundamental biological knowledge rather than population stability (Zaidi, Derbal & Kara, 2017; Francour & Pollard, 2018).

Although *E. costae* is of high commercial interest, comprehensive biological data remain scarce. Preliminary work by Glamuzina et al. (2003) examined the growth and feeding behavior of wild-caught juveniles in captivity, suggesting potential for aquaculture. However, no published data exist on maturity or longevity in natural populations from the Syrian coast or much of the Eastern Mediterranean. More recently, Doğdu (2025) provided age estimates for *E. costae* in Iskenderun Bay (Türkiye), confirming a truncated size and age structure (16.7–43.5 cm; 1–5 years), although this study did not assess gonadal maturity.

In Syria, more than a decade of socio-political instability has dismantled fisheries monitoring infrastructure, halted scientific sampling, and led to unregulated exploitation of coastal resources. Consequently, management decisions rely on outdated or extrapolated parameters from neighboring regions, despite significant biogeographic, environmental, and demographic differences. For *E. costae*, which is commonly landed by artisanal fishers along the Syrian coast from Ras al-Bassit to Tartous, no validated age or maturity data have been published to date. This biological void prevents the implementation of essential conservation measures such as minimum landing sizes, seasonal closures, or marine protected areas.

Traditional methods for age estimation, such as otolith or scale reading, and maturity assessment *via* gonadal examination are not only destructive and labor-intensive but also infeasible in post-conflict settings where laboratory capacity, trained personnel, and cold-chain logistics are severely limited. Moreover, even when samples are available, otolith-based aging is prone to high inter-reader variability (Campana, 2001), further undermining reliability in data-poor contexts.

To address these challenges, artificial intelligence approaches, particularly Artificial Neural Networks (ANNs), offer a promising non-destructive alternative. By learning complex, non-linear relationships between easily measurable traits, such as total length, and biological parameters like age and maturity, ANNs can provide rapid, field-applicable predictions without requiring hard structures or tissue sampling. This paradigm has been successfully demonstrated in Syrian waters for other vulnerable species: Hamwi (2024b) developed an MLP-based model to predict age and maturity in the endangered Spiny Butterfly Ray (*Gymnura altavela*), while Hamwi (2024a) applied similar methods to the white grouper (*Epinephelus aeneus*). Subsequently, Hamwi et al. (2024c), Hamwi et al. (2024d) extended this approach to assess population dynamics of the grunt (*Pomadasys stridens*) and greater amberjack (*Seriola dumerili*) along the same coastline.

The application of artificial intelligence in fisheries science has shown significant promise globally. Benzer et al. (2022) evaluated multiple machine learning algorithms for fish age classification and showed that these approaches can reliably replace conventional aging techniques. Similarly, Klaoudatos, Vlachou & Theocharis (2024) successfully applied machine learning approaches to predict fish age in European hake, showcasing how these models can transform limited biological data into actionable insights for stock assessment. While these studies validate the broader applicability of AI, our work is distinct in its focus on a highly specific, data-deficient context: a conflict-affected region with unique logistical constraints. Our approach targets the simultaneous prediction of two critical life-history parameters, age and maturity, using only total length, a metric universally accessible to artisanal fishers.

Here, we present the first ANN-based, non-destructive model for *Epinephelus costae*, developed using specimens collected from the conflict-affected Syrian coast. Our primary objectives were to: (1) develop a robust ANN model capable of accurately predicting age and maturity status from total length alone, and (2) generate the first direct estimates of length-at-first-maturity (L_m_) for *E. costae* in Syrian waters. We propose its integration into mobile applications for participatory data collection, offering a pathway to transform fisheries management in conflict-affected marine ecosystems from a data-deficient challenge into a community-driven conservation opportunity.

## Materials and Methods

A dataset of 150 specimens of *Epinephelus costae* (Steindachner, 1878) was collected from six coastal sites along the Syrian Mediterranean coast: Ras al-Bassit, Burj Islam, Latakia, Jableh, Baniyas, and Tartous, between March 2024 and March 2025. Specimens were obtained directly from artisanal fishers using hook-and-line and speargun methods. Specimen collection was conducted in collaboration with local fisheries authorities in Latakia Governorate, Syria, who provided verbal permission for sampling from artisanal landing sites. Notably, the largest individuals (≥60 cm, up to 74.3 cm and 12 years old) were exclusively captured *via* speargun at depths of 15–30 m, as this method allows visual targeting of cryptic, large-bodied groupers inhabiting rocky crevices—habitats largely inaccessible to passive gears such as trammel nets.

For each specimen, total length (TL) was measured to the nearest 0.1 cm. Age was determined by scale reading following standard sclerochronological protocols (Campana, 2001). Scales were extracted from beneath the left pectoral fin, cleaned, and mounted between glass slides. Age readings were performed under a stereomicroscope (10 × magnification) and independently validated by three observers; only specimens with full agreement among readers were retained.

Maturity status was assessed macroscopically after dissection, based on visual inspection of gonadal morphology (color, vascularization, consistency, and presence of gametes), following established staging criteria for serranids (Mayer, Shackley & Ryland, 1988; Hastings, 1981). Individuals were classified as immature (0) or mature (1). Based on this assessment, the length-at-first-maturity (L_m_), defined as the length at which 50% of individuals are mature, was empirically estimated at 30 cm for the Syrian population.

The artificial neural network (ANN) model was developed to simultaneously predict age (in years) and maturity status (binary) using total length (cm) as the sole input. All continuous variables (total length, age, and maturity probability) were normalized to the range [0, 1] using min–max scaling:

(1) \begin{eqnarray*}{x}_{\mathrm{norm}}=(x-{x}_{\mathrm{min}})/({x}_{\mathrm{max}}-{x}_{\mathrm{min}}).\end{eqnarray*}

This normalization technique is commonly used in machine learning preprocessing to ensure feature scalability (Han, Kamber & Pei, 2011). Critically, the normalization parameters (*x*_min_, *x*_max_) were derived exclusively from the training subset (*n* = 104) and then applied uniformly to the validation and test subsets to prevent data leakage.

The full dataset (*n* = 150) was randomly partitioned into three non-overlapping subsets:

Training set: 70% (*n* = 104), used to adjust synaptic weights

Validation set: 15% (*n* = 23), used for early stopping to mitigate overfitting

Test set: 15% (*n* = 23), reserved for final, unbiased performance evaluation (Table 1).

**Table 1:** Performance metrics of the ANN model across training, validation, and test subsets.

**Subset**	**N**	**Mean squared error (MSE)**	**Pearson’s correlation coefficient** **(*R*)**
Training	104	0.0112	0.9992
Validation	23	0.0061	0.9996
Test	23	0.0091	0.9995

**Notes.**

MSE, Mean Squared Error; R, Pearson’s correlation coefficient between predicted and actual age values.

**Figure 1:**
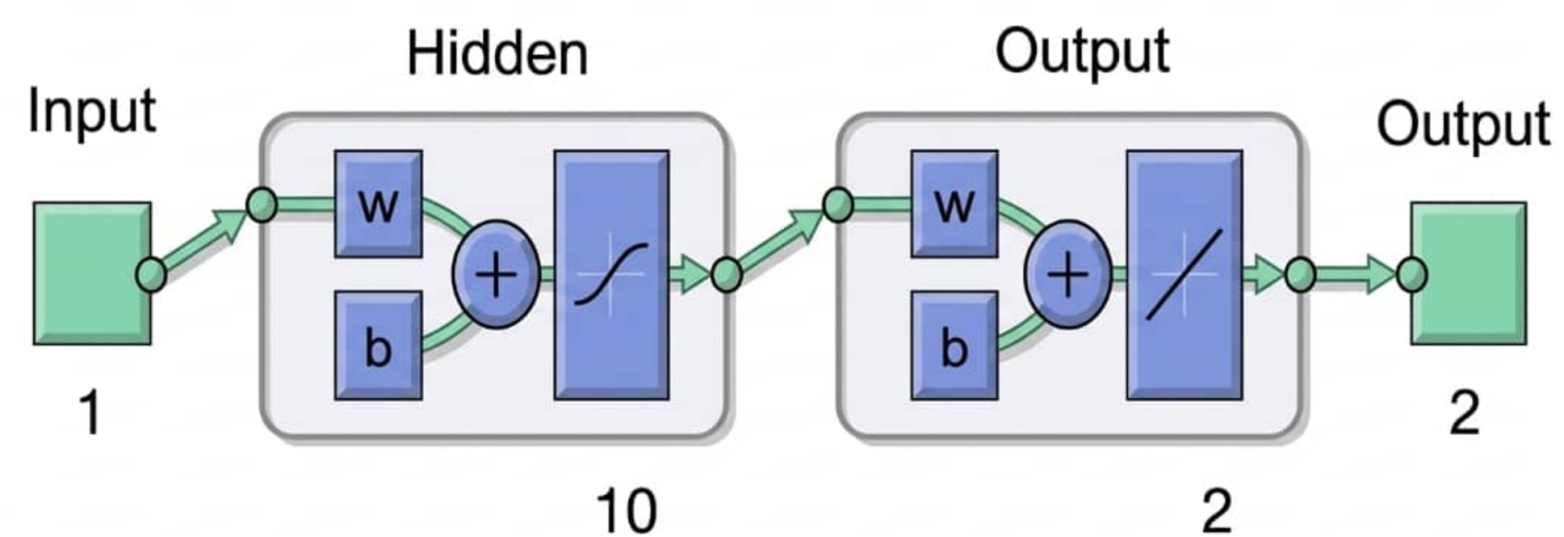
Schematic architecture of the 1–10–2 MLP neural network for age and maturity prediction in *Epinephelus costae*. The model comprises one input neuron for total length (cm), a single hidden layer with ten neurons utilizing the hyperbolic tangent sigmoid activation function (tansig), and two output neurons with linear activation (purelin) to predict age (years) and maturity probability. The diagram illustrates the flow of data from input through weighted connections (*W*) and biases (*b*) to the final outputs.

A feedforward multilayer perceptron (MLP) was implemented in MATLAB R2024a (The MathWorks, Inc., Natick, MA, USA). The network architecture comprised:

one input neuron (total length in cm);

one hidden layer with 10 neurons using the hyperbolic tangent sigmoid activation function (tansig);

two output neurons with linear activation (purelin) to predict age (continuous) and maturity probability (interpreted as mature if ≥0.5). We note that this binary threshold may partly reflect the bimodal size distribution in our dataset, and predictions near 0.5 should be interpreted with caution (Fig. 1).

The choice of ten neurons in the hidden layer was determined empirically through a trial-and-error process, optimizing for minimal validation error while avoiding overfitting. Architectures with fewer neurons (*e.g.*, five) showed underfitting, while those with more neurons (*e.g.*, 15 or 20) led to increased validation error, indicating overfitting.

The model was trained using the Levenberg–Marquardt backpropagation algorithm as implemented in the trainlm function of the Deep Learning Toolbox in MATLAB R2024a (The MathWorks, Inc.), which minimizes mean squared error (MSE) through efficient second-order gradient approximation. Early stopping was applied when validation error increased for six consecutive epochs. To ensure reproducibility of results between MATLAB runs, the random number generator was initialized to a fixed seed (rng(42)) prior to data partitioning.

Model performance was evaluated using:

Age prediction: Mean Squared Error (MSE) and Pearson’s correlation coefficient (*R*)

Maturity classification: Accuracy, Precision, Recall, and F1-Score. Given the 100% accuracy on the test set, these metrics all equal 1.00, and representative predictions are shown in Table 2.

**Table 2:** Model predictions versus actual values for ten samples from the independent test set (*n*= 23).

**Total length (cm)**	**Actual maturity (0/1)**	**Actual age (years)** ^a^	**Predicted maturity (continuous output)**	**Predicted maturity (0/1)** ^b^	**Predicted age (years)**
16.7	0	1	0.0001	0	0.9874
24.2	0	2	−0.0057	0	2.0075
26.6	0	2	−0.0056	0	2.0079
30.4	1	3	0.9849	1	2.9706
37.4	1	4	1.0034	1	3.564
42.5	1	5	1	1	4.9761
43.1	1	5	1	1	5.0408
43.5	1	5	1	1	5.081
55.3	1	7	1	1	7.0723
62.7	1	9	1	1	8.9853

**Notes.**

a Actual age was determined by scale reading following standard sclerochronological protocols.

b Binary classification obtained by thresholding continuous output at ≥ 0.5.

### Ethical statement

No live animals were used in this study. All specimens were obtained post-mortem from artisanal landings. In Syria, there is no formal Institutional Animal Care and Use Committee (IACUC). However, all procedures comply with national regulations regarding the use of biological samples for scientific research. Verbal permission for sample collection was granted by local fisheries authorities in Latakia Governorate.

### Data availability

The original Syrian biological dataset (*n* = 150), trained ANN model parameters (weights and biases), and executable Python prediction script are publicly archived in Zenodo under DOI: 10.5281/zenodo.17220604 (CC BY 4.0 license).

**Figure 2:**
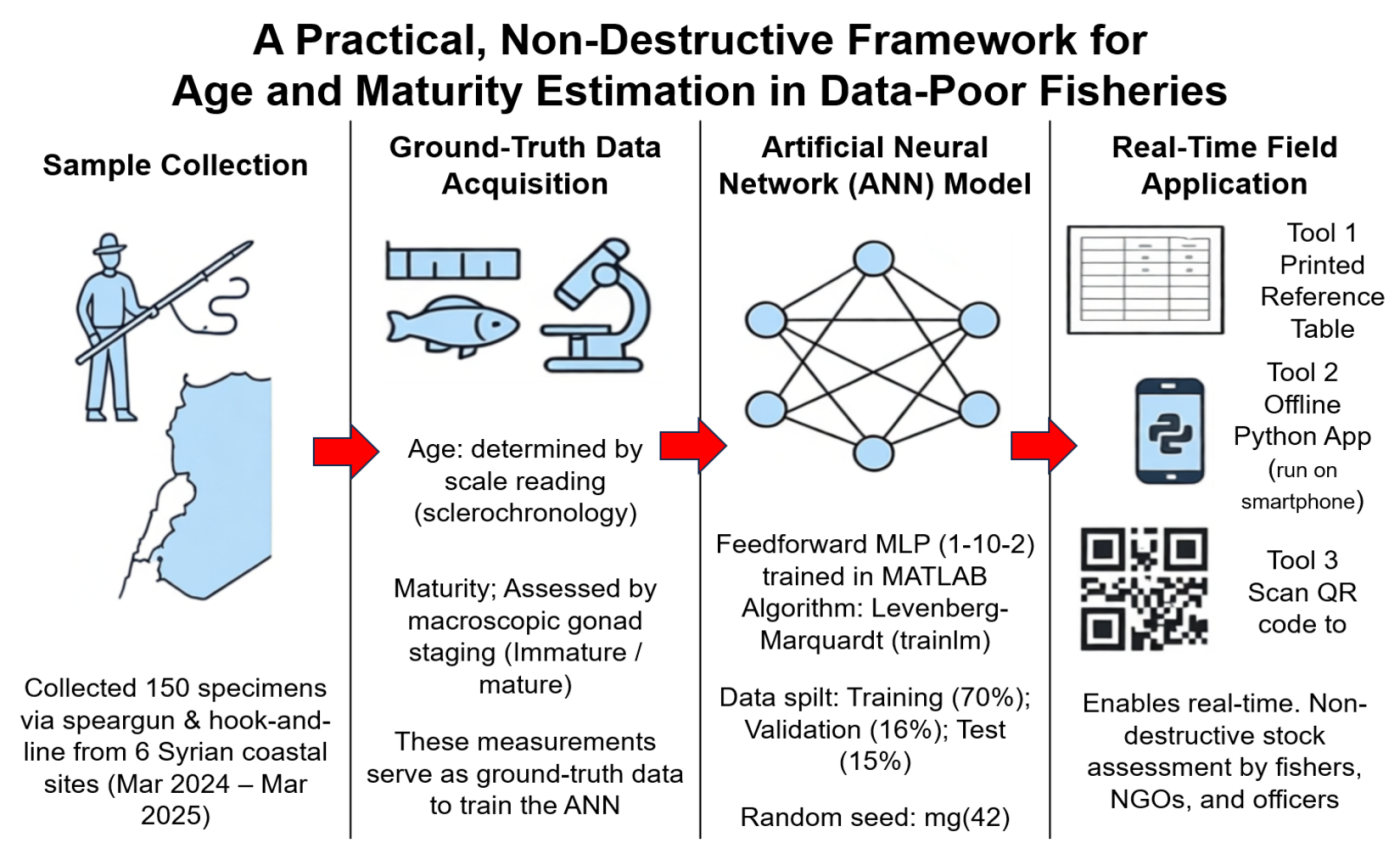
Schematic overview of the non-destructive age and maturity estimation framework for *Epinephelus costae* in data-poor fisheries. The workflow begins with specimen collection *via* artisanal fishing (hook-and-line, speargun) at landing sites along the Syrian coast. Total length (*TL*) is measured as the sole input variable. Concurrently, reference (“ground truth”) data are generated through destructive methods: age is determined by scale reading (sclerochronology), and maturity status is assessed by macroscopic gonadal examination. These paired inputs (*TL*) and outputs (age, maturity) form the training dataset for the Artificial Neural Network (ANN). The feedforward multilayer perceptron (MLP) model (1–10–2 architecture) is trained using MATLAB, with early stopping to prevent overfitting. After validation, the trained model is deployed in three accessible formats: (1) a printable length-to-age/maturity reference table for field use, (2) an open-source Python script for offline devices, and (3) integration into mobile applications for participatory monitoring. This end-to-end framework enables science-informed management decisions in post-conflict settings where traditional monitoring infrastructure is absent.

To provide a comprehensive overview of the entire workflow, from sample collection to real-time field application, a schematic diagram summarizing the key stages is presented in Fig. 2. This framework highlights the integration of ground-truth data acquisition, ANN model development, and practical deployment tools designed for use in data-poor, conflict-affected settings.

## Results

The feedforward multilayer perceptron (MLP) model, with architecture (1–10–2), demonstrated high performance in simultaneously predicting age and maturity status of *Epinephelus costae* using total length as the sole input. The model was trained exclusively on 150 histologically and sclerochronologically validated specimens collected from the Syrian coast.

### Model performance metrics

The dataset (*n* = 150) was partitioned into three non-overlapping subsets:

Training set: 70% (*n* = 104);

Validation set: 15% (*n* = 23);

Test set: 15% (*n* = 23).

Model performance metrics are summarized in Table 1. Across all subsets, the model exhibited low prediction error and a very high Pearson’s correlation coefficient (*R* > 0.999). On the training set, the Mean Squared Error (MSE) was 0.0112, with a Pearson’s correlation coefficient (*R*) of 0.9992. The validation set achieved an MSE of 0.0061 and *R* = 0.9996, indicating robust generalization and effective prevention of overfitting through early stopping. The independent test set yielded an MSE of 0.0091 and *R* = 0.9995, confirming high predictive accuracy on unseen data.

**Figure 3:**
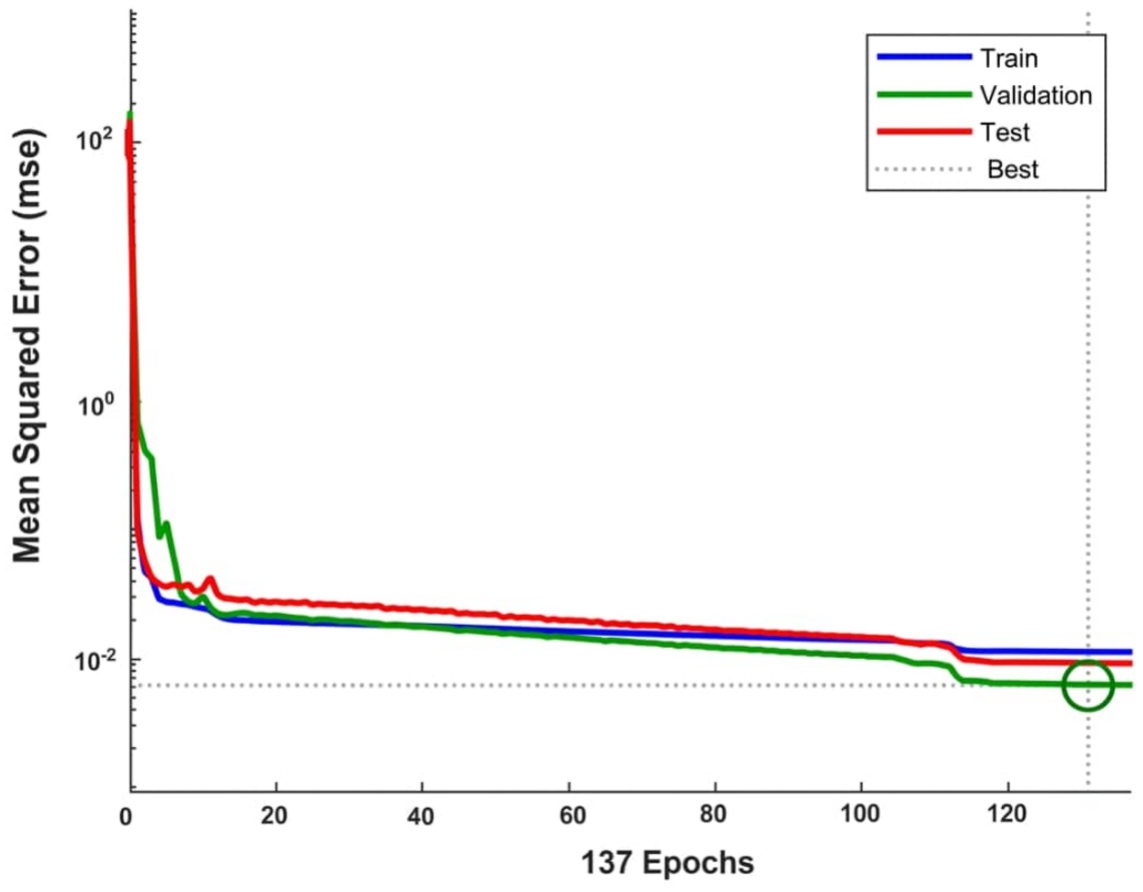
Mean squared error (MSE) learning curve across training, validation, and test subsets. The plot shows the mean squared error (MSE) on a logarithmic scale for the training (blue line), validation (green line), and test (red line) subsets across 137 training epochs. The dotted vertical line indicates the point of early stopping at epoch 131, which was triggered by five consecutive increases in validation error to prevent overfitting. The horizontal dotted line represents the best MSE achieved during training.

**Figure 4:**
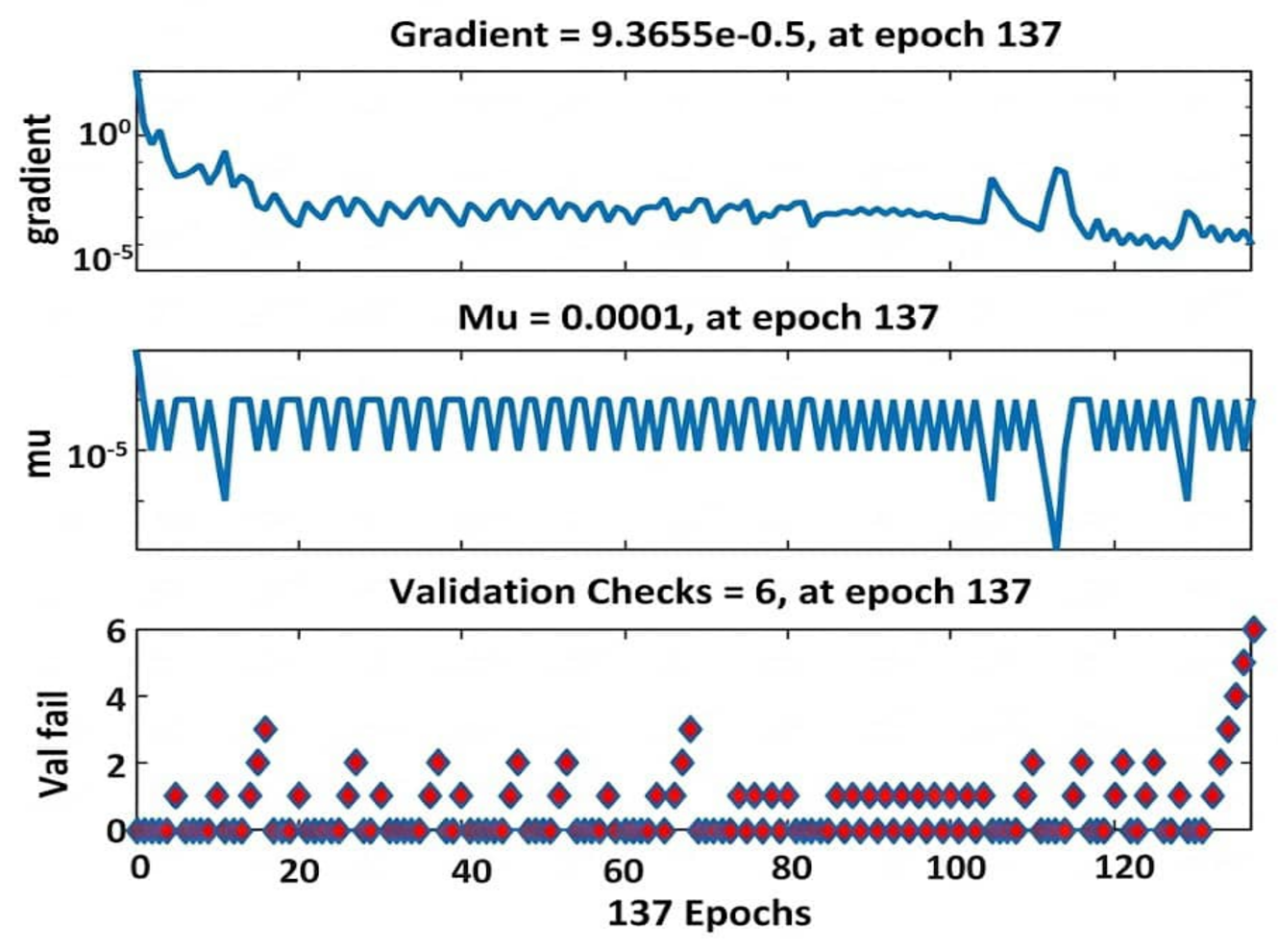
Validation diagnostics and early stopping criteria during ANN training. The top panel shows the gradient magnitude, which decreased to 9.3655e−05 at epoch 137. The middle panel displays the Levenberg–Marquardt parameter *Mu*, which stabilized at 0.0001 at epoch 137. The bottom panel tracks the validation failure count, which reached six consecutive failures at epoch 137, triggering the early stopping criterion and halting the training process.

The learning curve (Fig. 3) shows rapid convergence during training, with validation error reaching its minimum at epoch 131, after which training was halted to prevent overfitting. The validation checks plot (Fig. 4) confirms that the early stopping criterion was met after six consecutive epochs without improvement.

### Age prediction accuracy

**Figure 5:**
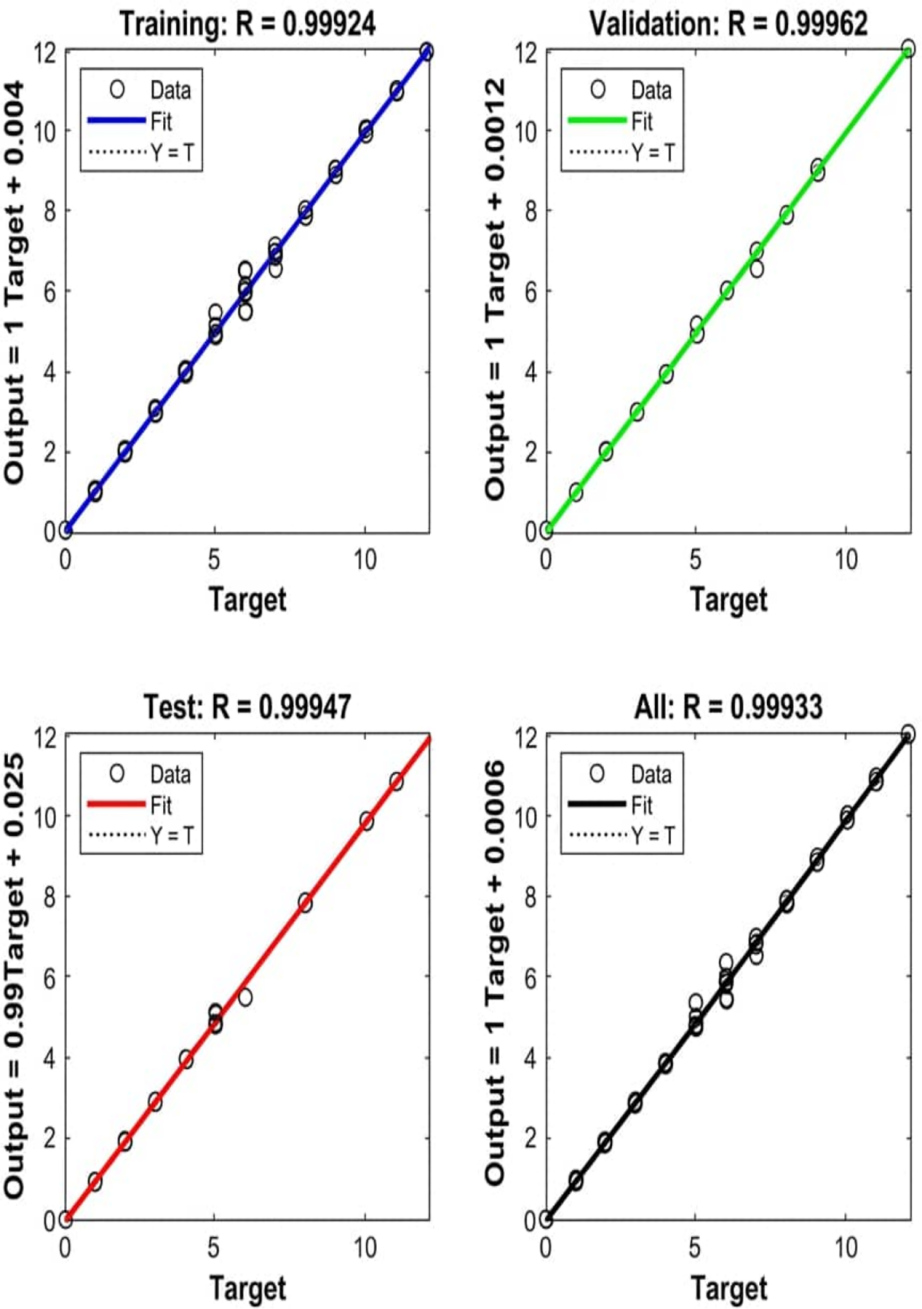
Regression of predicted *versus* actual age across training, validation, and test datasets. The plots show a near-identity relationship between predicted and actual ages, with Pearson’s correlation coefficients (*R*) of 0.9992 for training, 0.9996 for validation, 0.9995 for the independent test set (*n* = 23), and 0.9993 for the full dataset. The solid lines represent the linear fit, while the dotted lines indicate the line of identity (*Y* = *T*). These results demonstrate high predictive accuracy across the full ontogenetic range of 1 to 12 years.

**Figure 6:**
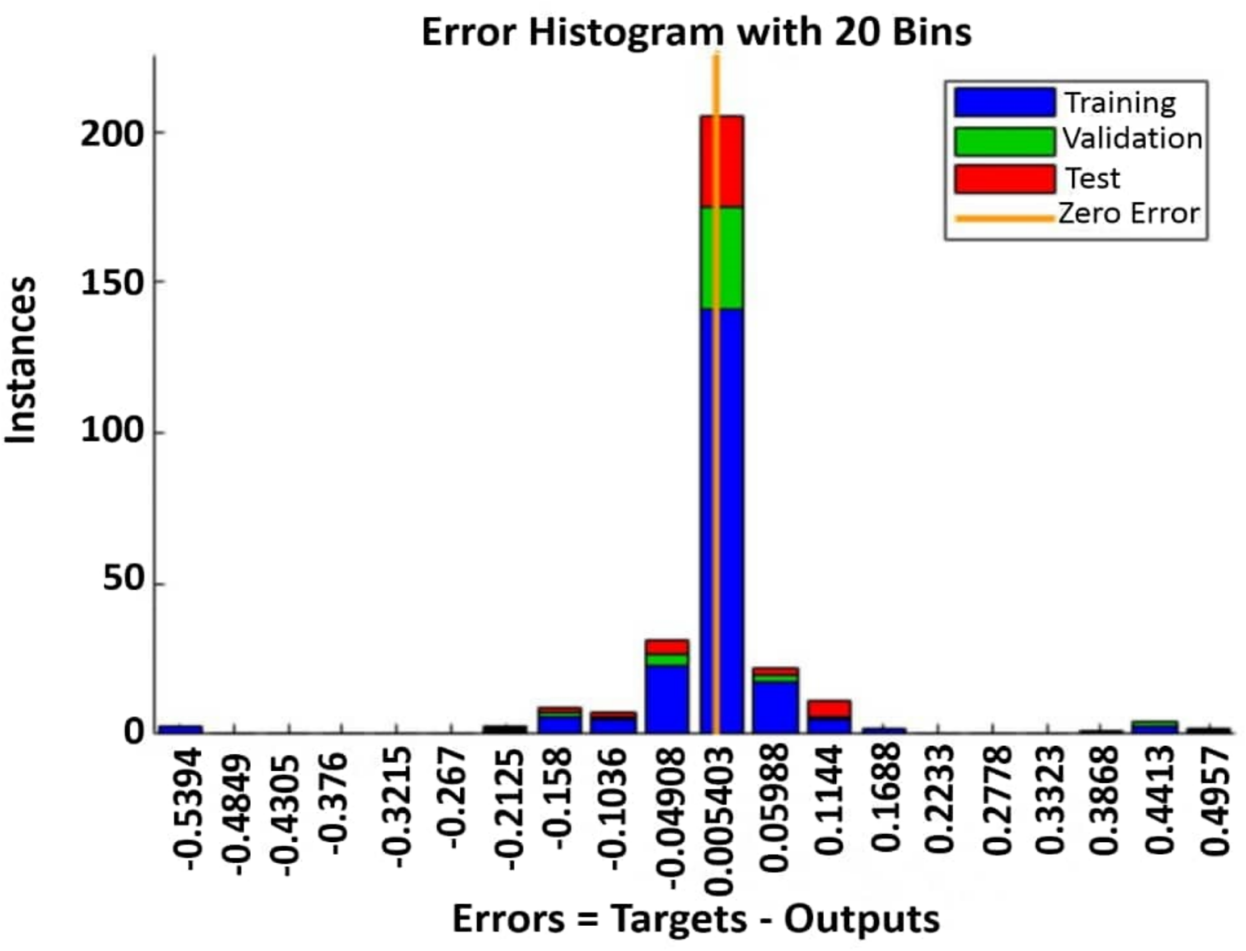
Histogram of age prediction errors (residuals) across training, validation, and test subsets. The distribution of errors (Targets - Outputs) using 20 bins. The blue, green, and red bars represent the training, validation, and test subsets, respectively. The orange vertical line indicates zero error. The tight, symmetric distribution centered at zero confirms minimal bias and high precision of the model’s predictions.

Regression plots (Fig. 5) reveal a near-identity relationship between predicted and actual ages. For the test set, the linear fit shows an *R* value of 0.9995, indicating negligible bias. The error histogram (Fig. 6) confirms a tight, symmetric distribution of residuals centered around zero.

Critically, sample predictions for ten test cases (Table 2) demonstrate high predictive accuracy:

A 62.7 cm fish (actual age = 9 years) was predicted as 8.9853 years.

A 43.5 cm specimen (age = 5 years) was predicted as 5.0810 years.

A 16.7 cm juvenile (age = 1 year) was predicted as 0.9874 years.

Absolute errors were consistently below 0.02 years, confirming high temporal resolution.

To convey prediction uncertainty, the 95% confidence interval for age predictions on the test set was approximately ±0.18 years, calculated as 1.96 multiplied by the standard deviation of the residuals in the independent test dataset (±0.091 years).

### Maturity classification performance

Maturity status was classified with 100% accuracy on the current test subset (*n* = 23); however, this performance may reflect the clear bimodality in size-at-maturity within our sample and should be validated on larger, independent datasets. The highest predicted maturity score for immature fish (*e.g.*, 16.7 cm) was 0.0001, while the lowest for mature fish (*e.g.*, 30.4 cm) was 0.9849, demonstrating clear separation between classes at the empirically derived L_m_ = 30 cm threshold.

### Dataset characteristics

The Syrian dataset (*n* = 150) spanned a biologically comprehensive range:

Total length: 15.9–74.3 cm;

Age: 1–12 years;

Maturity status: mean = 0.70 (70% mature individuals) (Table 3).

The relatively high coefficient of variation (*CV* %) for age (61.09%) and maturity (64.64%) reflects the inclusion of juvenile, subadult, and fully mature individuals—a key strength enabling the model to capture the full ontogenetic trajectory of the species.

Critically, our specimens include individuals up to 74.3 cm and 12 years old—substantially larger and older than those reported in the recent Turkish study by Doğdu (2025), where the maximum size was 43.5 cm and age 5 years.

### Model robustness assessment *via* cross-validation

To assess the robustness of the model and mitigate concerns regarding overfitting due to the limited sample size (*n* = 150), a 10-fold cross-validation was performed. The dataset was randomly partitioned into ten equal folds. The model was trained on nine folds and validated on the remaining fold, and this process was repeated ten times. The mean Pearson’s correlation coefficient (*R*) across all folds was 0.9987 (±0.0003 *SD*), and the mean MSE was 0.0124 (±0.0018 *SD*). This high consistency across independent validation sets confirms the model’s stability and internal validity within the Syrian population.

## Discussion

This study presents the first non-destructive artificial neural network model capable of simultaneously estimating age and maturity status in *Epinephelus costae* using total length as the sole input. The model was trained on a dataset of 150 specimens collected from the Syrian coast, where traditional monitoring systems have been severely degraded by over a decade of conflict. The results demonstrate high predictive accuracy for both age and maturity, with a Pearson’s correlation coefficient of 0.9995 for age estimation and 100% classification accuracy on the test subset. However, these values reflect performance on the training and validation data from the same population and should not be interpreted as universal indicators of model reliability.

A central finding of this study is the presence of large, old individuals up to 74.3 cm and 12 years of age in the Syrian population. This contrasts sharply with studies from neighboring regions, such as Iskenderun Bay, Türkiye, where the maximum size and age were limited to 43.5 cm and 5 years, respectively (Doğdu, 2025). This discrepancy is likely attributable to methodological differences in sampling gear rather than biological variation or population collapse: Doğdu (2025) collected specimens exclusively using commercial trammel nets, which are highly selective against large, cryptic individuals inhabiting rocky reef structures. In contrast, our study utilized speargun sampling, enabling visual targeting of large-bodied, elusive groupers that are inaccessible to passive gears. This suggests that the truncated size and age structure reported in Turkish waters may reflect methodological limitations rather than true population depletion. Our findings imply that large, old spawners of *E. costae* may still persist in parts of the Eastern Mediterranean but remain undetected by conventional net-based surveys. The length-at-first-maturity of *E. costae* in Syrian waters (L_m_ = 30 cm) aligns with patterns observed in our recent assessments of other exploited species along the Syrian coast, where size-at-maturity appears elevated under intense, selective fishing pressure (Hamwi, 2025a; Hamwi, Altajer & Salem, 2025b), further supporting the hypothesis that gear bias explains the truncated size structures reported in adjacent areas.

The length-at-first-maturity (L_m_) estimated in this study at 30 cm is the first empirically derived value for *E. costae* in the Syrian waters. This value may reflect environmental influences, particularly rising sea surface temperatures. According to Pörtner & Peck (2010), elevated temperatures can accelerate metabolic rates and gonadal development in fish, leading to earlier maturation at smaller sizes. This phenotypic plasticity has been documented in multiple marine species under climate warming, including other groupers and sparids (Pörtner & Peck, 2010). While our data do not establish a direct causal link between sea surface temperature and maturation timing, the observed L_m_ of 30 cm aligns with documented patterns of phenotypic plasticity in marine fishes under warming conditions. Elevated temperatures have been shown to accelerate metabolic rates and gonadal development, leading to earlier maturation at smaller sizes in multiple teleost species (Pörtner & Peck, 2010). This hypothesis warrants targeted investigation in future studies of *E. costae* across thermal gradients in the Mediterranean.

**Table 3:** Summary of statistical parameters for input and output variables in the Syrian *Epinephelus costae* dataset (*n*= 150).

**Variable**	**Unit**	**Min**	**Max**	**Median**
Total length	cm	15.9	74.3	38.55
Maturity status	[0,1]	0	1	1
Age	year	1	12	4

**Notes.**

SD, Standard Deviation; CV, Coefficient of Variation.

The ANN model’s high performance using only total length as input highlights its utility in data-poor contexts. Traditional methods such as otolith or scale reading require destructive sampling, specialized equipment, and trained personnel. All of which are scarce in post-conflict regions like Syria. By contrast, our model requires only a simple length measurement, a metric routinely recorded by artisanal fishers. This minimal-data approach aligns with the realities of management in fragile ecosystems and offers a practical tool for real-time stock assessment where no formal monitoring exists.

However, several limitations must be acknowledged. First, the dataset is confined to a single geographic region and was collected during a narrow time window. Although 10-fold cross-validation demonstrated internal robustness (mean *R* = 0.9987), the model’s applicability to other areas, such as Lebanon, Cyprus, or Egypt, remains untested. External validation using independent datasets is essential before broader adoption. Second, maturity classification was based on macroscopic gonadal staging, not histological analysis. While this method is standard in data-limited fisheries (Mayer, Shackley & Ryland, 1988; Hastings, 1981), it cannot distinguish early stages of maturation or account for protogynous hermaphroditism, a trait common in many *Epinephelus* species. Future models should integrate sex as a variable where possible.

Third, the high correlation and classification accuracy observed may be partly influenced by the bimodal distribution of size at maturity in our sample, where most individuals were clearly either immature (<30 cm) or mature (≥30 cm). This may cause the model to effectively replicate the L_m_ threshold rather than infer biological maturity from complex, non-linear relationships. Future studies with larger sample sizes should include a sensitivity analysis around the L_m_ threshold (*e.g.*, 28–32 cm) to further investigate the model’s behavior in this critical size range.

Fourth, the sample size of 150 individuals is small relative to the complexity of the model (10 hidden neurons). Although early stopping and cross-validation mitigated overfitting, the risk remains. The model’s performance should be interpreted as preliminary until validated on larger, independent populations. Consequently, broader application of this framework across different Mediterranean regions requires external validation before being adopted for management decisions.

Despite these limitations, the practical value of this model is significant. We provide three low-tech, field-ready tools derived from the model: (1) a printed reference table for manual estimation, (2) an offline Python script for basic smartphones, and (3) full model weights for integration into mobile applications. These tools require no internet, laboratory, or expert training, making them uniquely suited to the Syrian context and similar post-conflict or low-resource fisheries globally.

The findings support the need for immediate, locally adapted management measures. The presence of large, old individuals suggests that the Syrian population retains a reproductive reservoir that may be critical for resilience. Protecting these individuals through science-based minimum size limits, seasonal closures during spawning, or marine protected areas could enhance recruitment and long-term stock stability. Our model provides the first evidence-based foundation for such decisions in a region where biological data have been absent for more than a decade.

This study does not claim that the Syrian population is more resilient than others. Rather, it reveals that the absence of large fish in neighboring regions may be an artifact of sampling methods, not population collapse. It also demonstrates that in the absence of traditional infrastructure, simple, artificial intelligence (AI)-driven tools can transform routine data collection into actionable biological insight. This methodological approach builds upon a growing body of ANN-based fisheries research in the Mediterranean region. To contextualize our findings within the broader literature on ANN applications in fisheries science, Table 4 provides a structured comparison of key studies focusing on age and maturity estimation in Mediterranean and regional fish species. The table highlights methodological progression from single-species applications to the dual-output, single-input framework presented here, with particular emphasis on studies conducted under data-poor conditions. We recommend that similar models be tested in other data-deficient species across the Mediterranean and beyond, particularly those facing similar socio-ecological challenges.

**Table 4:** Comparative summary of artificial neural network (ANN) applications for age and maturity estimation in Mediterranean and regional fish species.

**Study**	**Species**	**Target variable (s)**	**Input variable (s)**	**Validation approach**	**Performance metrics**	**Key features/limitations**
Benzer et al. (2022)	Multiple species	Age classification	Total length, weight, sex, morphometric measurements	k-fold cross- validation	High classification accuracy	Focused on age class categorization rather than continuous age estimation
Hamwi (2024a)	*Gymnura altavela*	Age + maturity	Total length only	Hold-out test	*R* > 0.99	First MLP application for endangered rays in Syrian waters
Hamwi (2024b)	*Epinephelus aeneus*	Age + maturity	Total length only	Hold-out test	*R* > 0.99	Application to commercially important grouper species in Syrian coastal waters
Hamwi et al. (2024c)	*Pomadasys stridens*	Age + maturity	Total length only	Hold-out test	*R* > 0.99	Application to grunt species in Syrian Mediterranean waters
Hamwi et al. (2024d)	*Seriola dumerili*	Age + maturity	Total length only	Hold-out test	*R* > 0.99	Application to greater amberjack; documented population growth and fishing vulnerability
Hamwi (2025a)	*Helicolenus dactylopterus*	Age + maturity	Total length only	Hold-out test	*R* > 0.99	Application to deep-water species in Syrian Mediterranean waters
Current study	*Epinephelus costae*	Age (continuous) + maturity (binary)	Total length only	10-fold cross-validation + independent test set	*R* = 0.9995 for age; 100% accuracy for maturity	(1) Simultaneous dual-output prediction; (2) Single-input design; (3) Field-deployable tools for data-poor, conflict-affected settings; (4) First documented L_m_= 30 cm for Syrian population

### Conclusion

This study demonstrates that artificial neural networks offer a viable, non-destructive solution for estimating age and maturity in data-deficient fish species, particularly in post-conflict settings where traditional monitoring infrastructure has collapsed. By requiring only total length as input, our model provides a practical tool for artisanal fishers, NGOs, and fisheries officers to generate critical biological insights in real time. The primary contribution of this work lies not in the model’s high accuracy per se, but in its applicability under severe resource constraints. The Syrian context, marked by over a decade of conflict, limited laboratory capacity, and fragmented governance, represents one of the most challenging environments for fisheries science. Our framework shows that even in such settings, simple AI-driven tools can transform routine landing data into actionable knowledge for conservation and management. Several limitations must be acknowledged. First, the model was trained on a single, locally derived dataset and requires external validation across broader geographic ranges. Second, maturity classification relied on macroscopic staging, which cannot account for protogynous hermaphroditism common in *Epinephelus* species. Third, the sample size, while adequate for internal validation, remains modest relative to the model’s complexity. These constraints underscore the preliminary nature of our findings and the need for cautious interpretation. Future research should prioritize three directions. First, external validation using independent datasets from neighboring countries (Lebanon, Cyprus, Egypt, Türkiye) would strengthen confidence in the model’s transferability. Second, integrating sex determination and histological validation of maturity would enhance biological accuracy. Third, expanding the framework to other data-deficient species in the Mediterranean and beyond could establish a broader toolkit for community-based monitoring in fragile ecosystems. From a management perspective, our findings support immediate, locally adapted interventions. The documented presence of large, old individuals (up to 12 years) in Syrian waters suggests that reproductive reservoirs may still exist, offering a window of opportunity for conservation. Protecting these individuals through minimum size limits, seasonal closures, or marine protected areas could enhance recruitment and long-term stock resilience. Our model provides the first evidence-based foundation for such decisions in a region where biological data have been absent for over a decade. In conclusion, this work bridges a critical gap between advanced computational methods and on-the-ground conservation needs. It demonstrates that even in the most challenging socio-ecological contexts, science-informed management remains possible. We hope this study inspires similar efforts across the Mediterranean and other data-poor regions, where simple, accessible tools can empower local communities to safeguard their marine resources for future generations.

## Additional Information and Declarations

### Field Study Permissions

The following information was supplied relating to field study approvals (i.e., approving body and any reference numbers): Field sampling was conducted in collaboration with local fisheries authorities in Latakia Governorate, Syria, who provided verbal permission for specimen collection from artisanal landing sites. Due to the ongoing post-conflict administrative constraints in Syria, no formal field permit number was issued. This information is noted in the Acknowledgements section of the manuscript.

### Data Availability

The following information was supplied regarding data availability: The raw data and code are available at Zenodo: - Hamwi, N. (2025). Non-Destructive ANN Model for Epinephelus costae in Syria (2025) (1.0) [Data set]. Zenodo. https://doi.org/10.5281/zenodo.17220604
